# Ribosome profiling reveals the rhythmic liver translatome and circadian clock regulation by upstream open reading frames

**DOI:** 10.1101/gr.195404.115

**Published:** 2015-12

**Authors:** Peggy Janich, Alaaddin Bulak Arpat, Violeta Castelo-Szekely, Maykel Lopes, David Gatfield

**Affiliations:** 1 Center for Integrative Genomics, Génopode, University of Lausanne, 1015 Lausanne, Switzerland;; 2 Vital-IT, Swiss Institute of Bioinformatics, 1015 Lausanne, Switzerland

## Abstract

Mammalian gene expression displays widespread circadian oscillations. Rhythmic transcription underlies the core clock mechanism, but it cannot explain numerous observations made at the level of protein rhythmicity. We have used ribosome profiling in mouse liver to measure the translation of mRNAs into protein around the clock and at high temporal and nucleotide resolution. We discovered, transcriptome-wide, extensive rhythms in ribosome occupancy and identified a core set of approximately 150 mRNAs subject to particularly robust daily changes in translation efficiency. Cycling proteins produced from nonoscillating transcripts revealed thus-far-unknown rhythmic regulation associated with specific pathways (notably in iron metabolism, through the rhythmic translation of transcripts containing iron responsive elements), and indicated feedback to the rhythmic transcriptome through novel rhythmic transcription factors. Moreover, estimates of relative levels of core clock protein biosynthesis that we deduced from the data explained known features of the circadian clock better than did mRNA expression alone. Finally, we identified uORF translation as a novel regulatory mechanism within the clock circuitry. Consistent with the occurrence of translated uORFs in several core clock transcripts, loss-of-function of *Denr*, a known regulator of reinitiation after uORF usage and of ribosome recycling, led to circadian period shortening in cells. In summary, our data offer a framework for understanding the dynamics of translational regulation, circadian gene expression, and metabolic control in a solid mammalian organ.

The mammalian circadian system consists of a master pacemaker in the brain's suprachiasmatic nuclei (SCN) that synchronizes subsidiary oscillators present in most cell types. In the liver and other organs, up to 15% of gene expression shows daily oscillations that are driven directly by local clocks, or by systemic signals such as feeding and body-temperature rhythms (Vollmers et al. 2009; Mohawk et al. 2012; Zhang et al. 2014). Of the molecular mechanisms potentially accounting for rhythmic gene expression, transcription has been extensively studied, notably within the core clock circuitry consisting of transcriptional activators (mainly CLOCK; ARNTL/BMAL1; RORA, RORB, RORC) and repressors (mainly PER1, 2; CRY1, 2; NR1D1/REV-ERB alpha; NR1D2/REV-ERB beta). Their interactions in negative feedback loops generate transcriptional oscillations not only of clock genes, but genome-wide (Mohawk et al. 2012), which has led to the view that transcription represents the dominant driver of gene expression rhythms. However, post-transcriptional mechanisms likely contribute as well. In extension to earlier work showing poor overlap between liver proteome and transcriptome rhythms (Reddy et al. 2006), two recent studies have indicated that 20% (Robles et al. 2014) to 50% (Mauvoisin et al. 2014) of cyclically accumulating proteins are expressed from nonoscillating mRNAs. Conceivably, these protein rhythms are generated at the level of translation and/or protein stability. In other fields, translational regulation is emerging as key to understanding the overall moderate correlations between mRNA and protein abundances (Vogel and Marcotte 2012); a role in rhythmic gene expression is thus conceivable as well.

Time of day–dependent translation is not unprecedented in mammals. Transcripts encoding ribosomal proteins (RPs) associate with polysomes preferentially at the beginning of the night, coincident with feeding time (Jouffe et al. 2013). These mRNAs contain 5′-terminal oligopyrimidine (5′-TOP) motifs that are regulated by the nutrient-sensitive mammalian target of rapamycin complex 1 (TORC1) pathway (Meyuhas and Kahan 2015). Another documented mechanism for rhythmic translation involves daily dynamics in poly(A) tail length and the rhythmic activity of cytoplasmic polyadenylation element-binding proteins (CPEBs) (Kojima et al. 2012). Despite such individual examples, a comprehensive and quantitative analysis of rhythmic translation from a mammalian organ is still lacking.

We have used ribosome profiling (RPF-seq), a method based on the massively parallel sequencing of ribosome-protected mRNA footprints (Ingolia et al. 2009), to determine the positions of translating ribosomes transcriptome-wide and to establish a quantitative, high-resolution map of the mouse liver translatome around the clock. From RPF-seq and matching whole-transcriptome sequencing (RNA-seq) data, we determined the relationship between translation and mRNA abundance rhythms, uncovered the set of mRNAs for which these rhythms are uncoupled, and calculated translation efficiencies transcriptome-wide. Moreover, we inferred the relative levels of clock protein biosynthesis and identified upstream open reading frame (uORF) translation as a novel regulatory mechanism within the clock circuitry. Altogether, our study reveals key features of rhythmic protein biosynthesis and the impact of translational control on gene expression in a solid, highly differentiated mammalian organ with well-studied functions.

## Results

### Ribosome profiling in liver around the clock

**Figure 1.**
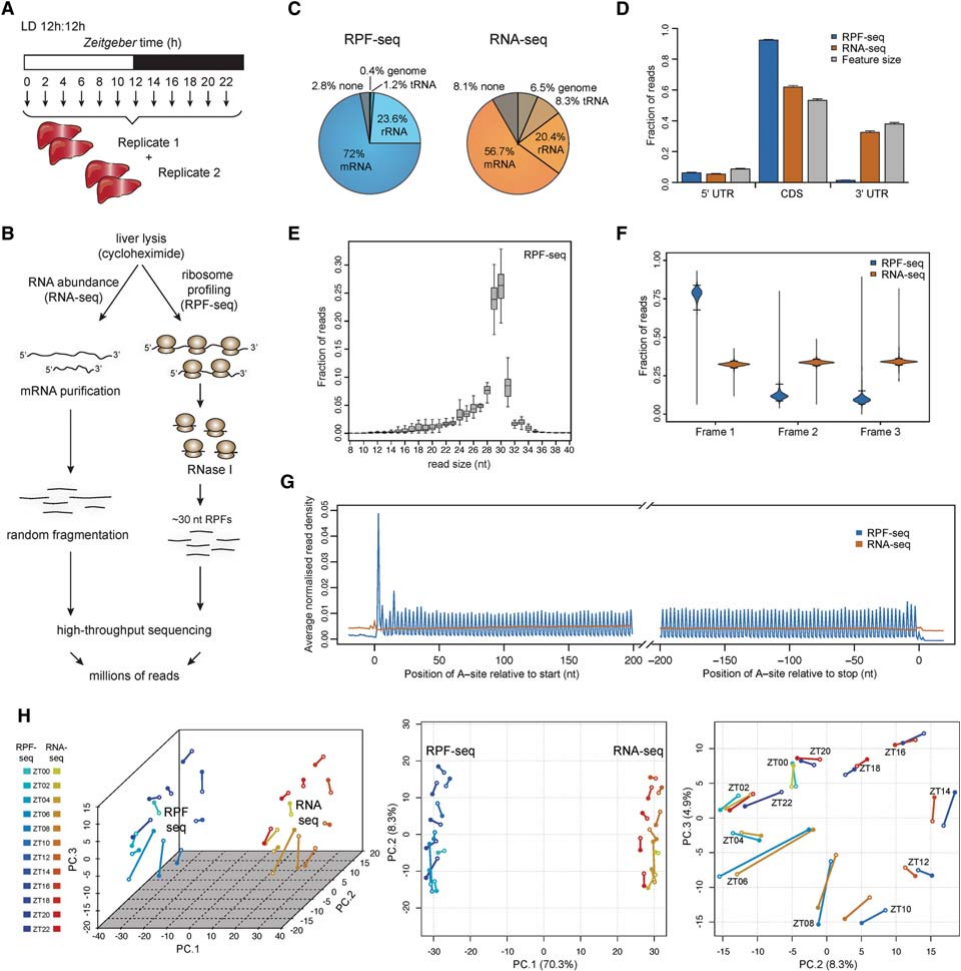
Time-resolved ribosome profiling data from mouse liver. (*A*) Overview of the experimental design for liver sampling over the 24-h cycle of the day. Forty-eight livers were collected and assembled into two replicate time series of 12 timepoints around the clock (each sample representing a pool of two mice). (*B*) Overview of the main steps in the protocol for the preparation of RNA abundance (RNA-seq) and ribosome profiling (RPF-seq) data; for details, see Supplemental Material. (*C*) Mapping summary of RPF-seq and RNA-seq reads across all replicates and timepoints. Note that RPF-seq reads were enriched for mRNAs, as expected. For detailed mapping outcome, see Supplemental Table S1. (*D*) Read distribution within 5′ UTRs, CDS, and 3′ UTRs for RPF-seq (blue) and RNA-seq data (orange) compared with the distribution expected by chance, which is determined by the feature sizes (gray; *N* = 10829). Note the enrichment of RPF reads within CDS, the depletion from 3′ UTRs, and considerable amounts of reads (6%) within the 5′ UTR. (*E*) Insert size distribution of RPF-seq reads across all replicates and timepoints shows that the majority of footprints are 29–30 nt in length. Box-and-whisker plots: midline, median; box, 25th and 75th percentiles. Whiskers extend to the minimum and maximum values within 1.5 times the interquartile range from the box. (*F*) Frame analysis for RPF-seq and RNA-seq reads within the CDS (using genes for which the expressed transcript isoforms define one main translated CDS/protein—called single protein isoform genes—with an RPF-RPKM [reads per kilobase per million mapped reads] value >5 and fulfilled a few other minor criteria described in Supplemental Material; *N* = 3793). RPF-seq reads show a clear preference for reading frame 1 (the annotated frame), whereas RNA-seq reads distribute equally across the three reading frames, as expected. Violin plots extend to the range of the data, with horizontal lines marking the 2.5% and 97.5% quantiles. (*G*) Read density distribution of RPF-seq and RNA-seq reads within 200 nt from the start or −200 nt from the stop codons reveals a 3-nt periodicity of RPF reads within coding sequences. The analysis used only transcripts from single protein isoform genes (see *F*) with RPF-RPKM > 5 and CDS > 400 nt (*N* = 3237) and quantified the number of reads per nucleotide based on the A-site prediction as described in the Supplemental Material. (*H*) Principal component (PC) analysis of RPF-seq and RNA-seq data sets, using the top-ranked 4000 genes (see Supplemental Methods). The first three PCs explain 70.3%, 8.3%, and 4.9% of total variation, respectively (3D scatter plot, *left* panel). While PC1 mainly reflected variance attributable to differences between the mRNA abundance and footprint data sets (*middle* panel), PC2 and PC3 resolved mainly variance attributable to factor time (*right* panel). Note that the timepoints assemble to a near-perfect “clock” in the PC2 versus PC3 representation. A scree plot showing contributions of further PCs can be found in Supplemental Figure S1D.

We collected time-resolved ribosome profiling data from 48 male mice entrained to light-dark cycles and euthanized at 2-h intervals around the clock. We assembled liver extracts into two independent replicate time series (12 timepoints, *Zeitgeber* time ZT0 to 22) (Fig. 1A), prepared ribosome footprints and matching total RNA, converted them into sequenceable libraries (Fig. 1B), and sequenced them with high coverage (Supplemental Table S1). Our protocol yielded high-quality footprints that mainly mapped to protein coding sequences (CDS) and were depleted from untranslated regions (UTRs) of mRNAs (Fig. 1C,D; Supplemental Table S1). Moreover, the predominant footprint length of 29–30 nt (Fig. 1E) allowed the precise identification of translated codons. The alignment of CDS-mapping reads relative to the position of the ribosome's aminoacyl tRNA-site (A-site; inferable from footprint length and sequence) (Ingolia et al. 2011) thus revealed excellent reading frame preference (Fig. 1F) and captured the CDS triplet codon composition transcriptome-wide (Fig. 1G). These characteristics were absent in the RNA-seq data, as expected (Fig. 1D–G; Supplemental Fig. S1A). Moreover, the quantification of CDS-mapping reads showed high reproducibility across biological replicates (Supplemental Fig. S1B,C). Finally, principal component analysis (PCA) on the ensemble of data sets (Fig. 1H) separated RPF-seq and RNA-seq data on PC1 and recapitulated its cyclic nature with near-perfect temporal resolution (PC2 and PC3); i.e., the covariates in the experimental design (RNA/RPF and factor time) were retrieved by an unsupervised method for sample clustering. We concluded that the data sets were of high technical quality and would be suitable for comprehensive analyses of rhythmic and constitutive translation in the liver.

### Hallmarks of translational regulation in liver

Ribosome profiling from mammalian tissues is still relatively uncommon, and we therefore started with a characterization of general properties of the translatome data, independently of its time resolution. From the ratio of CDS-mapping RPF-seq to RNA-seq reads, we first computed relative ribosome occupancies, which can be interpreted as relative translational efficiencies (TEs) because each footprint reflects the synthesis of an individual protein molecule and, importantly, integrating read numbers across the entire CDS corrects for local variation in footprint density (Ingolia et al. 2011). Briefly, while the local speed of translation elongation may vary (e.g., ribosome pausing due to RNA structure or codon usage) and represents a source of inhomogeneous footprint distribution on a given CDS, average translation speeds across genes appear to be rather constant (Ingolia et al. 2011). It should be noted, however, that a possible influence of local variation on overall translation speed of an mRNA has been suggested (Dana and Tuller 2012) and is a current topic of debate (Ingolia 2014).

**Figure 2.**
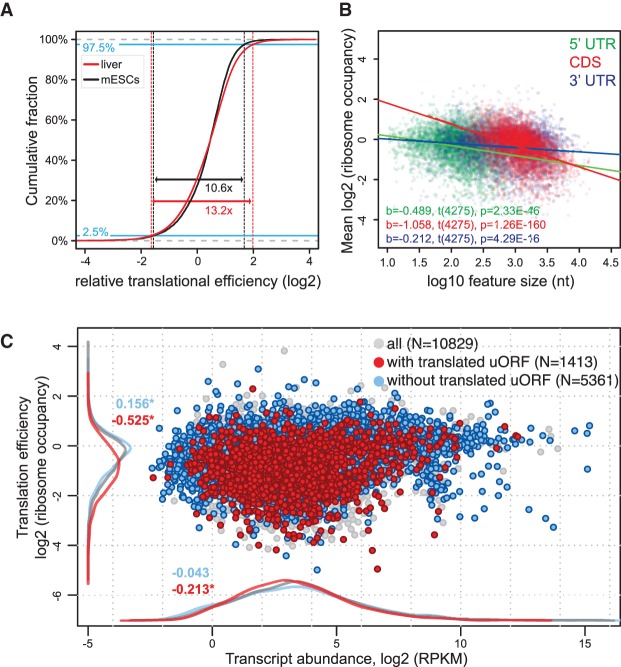
Analysis of translation efficiencies in mouse liver. (*A*) TE distribution in mouse liver (red curve; representing all 24 ZTs from 10829 genes, i.e., *N* = 129870 individual data points) compared with that in mESCs (black curve; data from Ingolia et al. 2011; *N* = 10217 genes). Liver data were adjusted for mean to ensure comparability with mESC data. The asymmetrical nature of the TE distribution that has previously been reported for mESCs and that is indicative of an intrinsic upper limit to translation rates (Ingolia et al. 2011) is also observed in the liver. Note that the TE range is significantly broader in the liver, where 95% of data fall into a 13.2-fold TE range, compared with a 10.6-fold range in mESCs (*P* < 2 × 10^−5^, permutation test). (*B*) Correlation between TEs and 5′ UTR, CDS, or 3′ UTR lengths. Analysis was performed on transcripts from genes for which the transcriptomics analysis showed that a single protein isoform was produced, and that had an RNA-seq RPKM value >5, and 5′ and 3′ UTR lengths ≥10 nt (*N* = 4277). Linear regression lines for each group are plotted *over* the data points, and related *t*-test results of the regression slopes are reported in the plot area with the same color code. Inverse and statistically significant correlation between TE and feature length was thus apparent for all three features, with predictive value CDS > 5′ UTR > 3′ UTR. (*C*) Scatter plot of TEs (ordinate) versus transcript abundances (TAs; abscissa) averaged over timepoints and replicates. Highlighted are transcripts from single protein isoform genes, which do (red) or do not (blue) contain at least one translated AUG-initiated uORF. Density curves of TEs and TAs for highlighted data points are plotted on the margins with same color code. uORF translation is thus associated with a pronounced TE decrease and a slight decrease in transcript abundances. Numbers on density curves reflect the location shift (log_2_ values of the median calculated from the differences across all timepoints) relative to all transcripts. Transcripts with translated uORF: TE, *P* < 2.2 × 10^−16^; TA, *P* = 1.3 × 10^−5^ (Wilcoxon rank-sum test). Transcripts without translated uORF: TE, *P* < 2.2 × 10^−16^; TA, *P* = 0.287 (Wilcoxon rank-sum test).

In mouse embryonic stem cells (mESCs), TEs cover an approximately 10-fold range and have an asymmetric distribution indicative of an intrinsic upper limit that is transcript-specifically decreased by inhibitory mechanisms (Ingolia et al. 2011). TEs in liver showed a broader dynamic range and analogous asymmetry (Fig. 2A). While multiple mechanisms are likely involved in establishing transcript-specific ribosome occupancies, several simple transcript features have previously been observed to correlate with TEs. In yeast, ORF length and translation rate correlate inversely, presumably due to a selection for faster translation initiation on loci encoding short proteins (Arava et al. 2003; Ingolia et al. 2009). Also in the liver data set, CDS lengths explained a significant proportion of variance in ribosome occupancies (*R*^2^= 0.16; *P* = 1.26 × 10^−160^) (Fig. 2B). 5′ UTR (*R*^2^= 0.047; *P* = 2.33 × 10^−46^) and 3′ UTR lengths (*R*^2^= 0.015; *P* = 4.29 × 10^−16^) correlated with TEs as well (Fig. 2B), and the predictive power of the 5′ UTR length remained significant even after correction for interdependence of UTR and CDS lengths (Supplemental Fig. S2A–C). These results are consistent with a prominent role for 5′ UTRs in translational control and with the idea that translation regulatory elements—of which longer 5′ UTRs potentially contain more—are predominantly inhibitory.

Within 5′ UTRs, uORFs are emerging as important *cis*-regulatory elements that control CDS translation, usually in an inhibitory fashion (Wethmar et al. 2014). Six percent of RPF-seq reads fell into annotated 5′ UTRs (Fig. 1C), albeit with pronounced transcript-specific variability (Supplemental Fig. S3A), suggestive of abundant uORF usage in the liver. uORFs are short and often poorly conserved (Churbanov et al. 2005) and frequently initiate at near-cognate (non-AUG) start codons (Ingolia et al. 2011), complicating prediction just from sequence. To explore uORF usage in the liver, we therefore compiled a uORF-enriched transcript set based on whether the 5′ UTRs harbored sequence stretches (1) embraced by AUG initiation and stop codons and (2) covered by footprints with distinct reading frame preference. Despite these simple criteria that miss, for example, non-AUG-initiated uORFs, the detected transcripts showed significantly lower main ORF TEs (difference in location of red vs. gray densities on the ordinate of −0.525 corresponds to >30% TE reduction; *P* = 2.2 × 10^−16^; Wilcoxon rank-sum test) (Fig. 2C), whereas the TEs of transcripts lacking translated uORFs were slightly increased (Fig. 2C, blue). Importantly, the correlation of low TEs with translated uORFs was independent of 5′ UTR (or CDS/3′ UTR) lengths (Supplemental Fig. S2D–F). Instead, linear regression analysis uncovered that the underlying cause for the correlation of TE with 5′ UTR length (see above) (Fig. 2B) was uORF presence rather than 5′ UTR length per se (Supplemental Fig. S2, cf. G–K and A–C). Finally, uORF-containing mRNAs were globally less abundant (Fig. 2C, red density on abscissa), possibly reflecting the activity of the nonsense-mediated mRNA decay (NMD) pathway that selectively degrades mRNAs with premature termination codons and that is known to act on uORF-containing transcripts (Mendell et al. 2004). We concluded that uORF usage is frequent in the liver and likely a major determinant of transcript-specific TEs, correlating with reduced protein biosynthesis from the CDS. Of note, we also analyzed another feature that may have been expected to correlate with TE, i.e., the presence of pause sites. By using an analogous approach to Ingolia et al. (2011) to identify local variation in footprint density that exceeded the CDS median (more precisely, we used the similar, but for low translated transcripts, less stochastic “trimean”) by a certain threshold, we found that the presence of such sites had no predictive power for translation rates (Supplemental Fig. S4A,B).

### In the core clock and globally, rhythmic mRNA abundance is a good predictor of footprint rhythms

**Figure 3.**
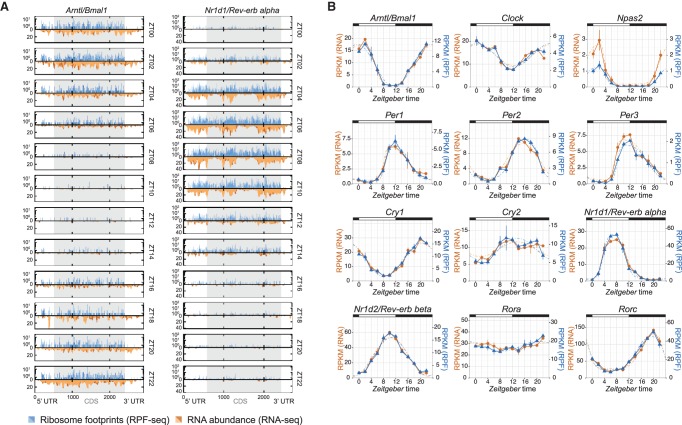
Core clock transcripts show mRNA abundance and ribosome occupancy in sync. (*A*) Time-resolved distribution (ZT00 to ZT22; arranged vertically) of normalized counts of ribosome profiling (RPF-seq; blue) and RNA abundance (RNA-seq; orange) reads along the *Arntl/Bmal1* and *Nr1d1/Rev-erb alpha* transcripts. Different color shadings (dark/light orange and blue) indicate the two biological replicates. Gray shading of the boxes marks the CDS; UTRs are in white. (*B*) RPKM values of CDS-mapping RPF-seq (blue) and RNA-seq (orange) data for circadian core clock genes around the 24-h daily cycle. Means per timepoint are plotted; error bars, the two biological replicates. Dashed lines represent rhythmic curve fittings to the data.

**Figure 4.**
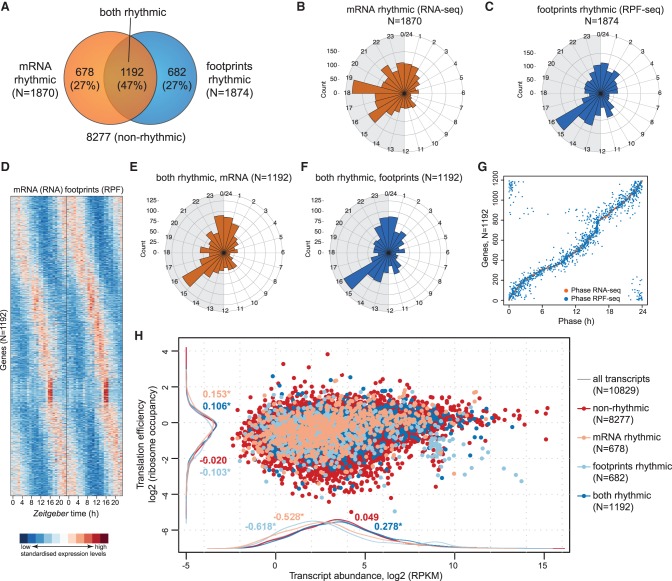
Transcriptome-wide analysis of transcript abundance and ribosome occupancy rhythms. (*A*) Venn diagram summarizing the result of rhythmicity detection in the RNA-seq and RPF-seq data. Of a total of 10,829 expressed protein-coding loci, 1870 showed mRNA rhythms and 1874 had footprint rhythms (in both cases 17% of all) with a >1.5× peak-to-trough amplitude (FDR < 0.05); 1192 transcripts were common to both sets. (*B*) Phase histograms for the transcripts in *A*, showing the peak phase distribution of mRNA abundance (RNA-seq) rhythms over the 24-h cycle in orange. The length of the spoke indicates how many transcripts peaked at a specific time. (*C*) As in *B* for footprint rhythm (RPF-seq) in blue. Note the different phase distribution of ribosome occupancy rhythms compared with RNA abundance rhythms depicted in *B*. (*D*) Heat map of rhythms at the level of mRNA abundance (RNA-seq; *left*) and footprints (RPF-seq; *right*) for the overlapping set from *A* (1192 genes). Transcripts are sorted by the phase of maximal ribosome occupancy. For the representation, mRNA abundances and translation levels are standardized within each gene (row) and independently for RNA-seq and RPF-seq columns (*Z*-scores). (*E*) Phase histograms showing the phase distribution of “mRNA and footprints rhythmic” transcripts from the overlap in *A* (*N* = 1192) for mRNA accumulation (RNA-seq) in orange. (*F*) As in *E*, but for footprints (RPF-seq) in blue. Note that the distribution is near-identical to that in *E*. (*G*) Phase correlation plot of the “mRNA and footprints rhythmic” genes (*N* = 1192). Each row contains two dots marking the phase of maximal mRNA abundance (orange) and the phase of maximal footprints (blue) for each gene. Genes are ordered according to the phase of the mRNA. (*H*) Scatter plot of TE versus transcript abundances for the genes classified into the different rhythmicity categories shown in *A* (values averaged over timepoints and replicates). Density curves are plotted on the margins with the same color code. Numbers on density curves reflect the location shift (log_2_ values) relative to all transcripts (gray). (*) Significance at the 0.05 level (Wilcoxon rank-sum test). The plot shows that rhythmic genes (i.e., those with rhythmic mRNAs [light salmon], with rhythmic footprints [light blue], or with rhythmic mRNA and footprints [dark blue]) are significantly different at the level of TE and of transcript abundances than transcripts of nonrhythmic genes (red).

We used the clock genes for first temporal analyses of the data sets. As illustrated by the anti-phasic expression of *Arntl* and *Nr1d1*, core clock transcripts were detected with high coverage and oscillated in both the RNA-seq and RPF-seq data sets (Fig. 3A). Read count integration over the CDS indicated that for all core clock components, footprint profiles closely matched mRNA abundance rhythms (Fig. 3B). We concluded that the rhythmic biosynthesis of core clock proteins was determined by mRNA availability with no further regulation by time of day–dependent translation (Supplemental Fig. S5A). We next conducted transcriptome-wide rhythmicity analyses. Applying a more than 1.5-fold peak-to-trough amplitude cut-off, we identified oscillations in the RNA-seq and RPF-seq data sets that affected in both cases ∼17% of the protein-coding transcriptome (almost 1900 mRNAs) (Fig. 4A; Supplemental Table S2). However, mRNA abundance and ribosome occupancy rhythms showed different peak phase distributions (Fig. 4B,C). In good agreement with previous reports (Le Martelot et al. 2012; Zhang et al. 2014), a majority of mRNAs thus showed maximal abundance during the night, with an enrichment around ZT15–ZT19 (Fig. 4B). In contrast, maximal translation was prevalent at the beginning of the dark phase, with a dominant peak around ZT15–ZT16 (Fig. 4C). These different distributions resulted from transcripts that were unique to either data set rather than from phase delays occurring between mRNA accumulation and translation, because the intersecting set of 1192 “mRNA and footprints rhythmic” transcripts (Fig. 4A) showed near-identical RNA-seq and RPF-seq oscillations (Fig. 4D–G; Supplemental Fig. S6A). We concluded that whenever both mRNA abundance and ribosome occupancy cycled, they globally did so in sync. Similar to the core clock components (Fig. 3), most rhythmic mRNAs were thus translated concomitant with their cellular accumulation and had constant TEs. Distinct out-of-phase translation was indeed confined to rather few exceptional cases (Supplemental Fig. S6B). Finally, it is noteworthy that the TEs of “mRNA and footprints rhythmic” transcripts were slightly increased compared with the global population of expressed transcripts (location shift of dark blue vs. gray densities of 0.106 corresponds to ∼8% higher TEs; *P* = 2.2 × 10^−05^; Wilcoxon rank-sum test) (Fig. 4H). No enrichment or depletion for rhythmic genes was seen with regard to AUG-initiated uORFs or pause sites (*P* = 0.533 and *P* = 0.315, respectively; Fisher's exact test).

### Widespread time of day–dependent translation of nonrhythmic mRNAs

**Figure 5.**
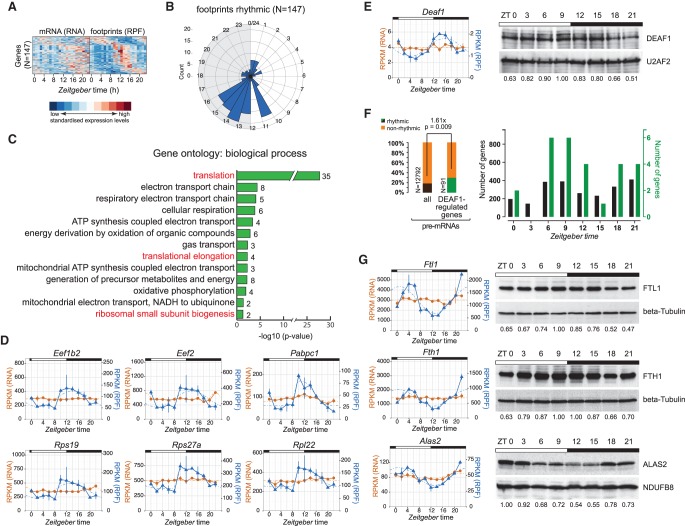
Daytime-dependent translational control of protein biosynthesis machinery components, of transcription factors, and in iron metabolism. (*A*) Heat map of “mRNA flat–footprints rhythmic” genes identified after *Babel* analysis (significant changes in ribosome occupancy) showing the mRNA abundance (RNA-seq; *left*) and footprint (RPF-seq; *right*) data for 147 genes. Transcripts are sorted by the phase of maximal ribosome occupancy. For the representation, mRNA abundances and translation levels are standardized within each gene (row) and independently for RNA-seq and RPF-seq columns (*Z*-scores). Note that this high-confidence set of 147 transcripts has clear rhythms at the translational level, but not so at the mRNA abundance level. (*B*) Phase histogram showing the phase distribution of footprint data across the 24-h cycle. Generally, there was a strong enrichment of ribosome occupancy maxima at the light-to-dark transition. This distribution is significantly nonrandom (*P* = 1.12 × 10^−10^; *R* = 0.395; Rayleigh uniformity test) and significantly different from those in Figure 4C (*P* < 7.6 × 10^−08^; *W* = 32.80; Watson–Wheeler test for homogeneity of angles). (*C*) Gene ontology (GO) analysis of biological process based on genes detected in *A*. Bar graph shows log_10_ of *P*-values for each category. Numbers *next* to the bars represent the number of genes within each category. Marked in red are the GO terms connected to the protein biosynthesis machinery. (*D*) RPF-seq (blue) and RNA-seq (orange) profiles for several rhythmically translated transcripts connected to the protein biosynthesis machinery—ribosomal proteins, translation factors, and poly(A) binding proteins—around the 24-h cycle. Means per timepoint are plotted; error bars, replicates. Dashed lines represent rhythmic curve fittings. (*E*, *left*) RNA-seq (orange) and RPF-seq (blue) profile as in *D* for transcription factor *Deaf1* around the clock. (*Right*) Western blot analysis for DEAF1 (and loading control U2AF2) in liver nuclear extracts. Numbers *below* the panels show relative levels of DEAF1 protein after normalization to U2AF2. Data from one representative time series are shown (*N* = 2). (*F*, *left*) Rhythmically transcribed genes (from Du et al. 2014) are 1.6-fold enriched in DEAF1-regulated genes (Fisher's exact test *P* = 0.009) (Yip et al. 2009), consistent with the idea that rhythmic translation and protein accumulation of DEAF1 leads to rhythmic transcriptional activity on its target genes. (*Right*) Peak abundances of rhythmically transcribed genes around the clock. (Black) All rhythmically transcribed genes; (green) rhythmic DEAF1-regulated genes. (*G*, *left*) RNA-seq and RPF-seq profiles for *Ftl1*, *Fth1*, and *Alas2*, similar as in *D*. Note the high-amplitude rhythms in translation from flat mRNAs. (*Right*) Confirmation of protein rhythmicity by Western blot analysis of total protein extracts (FTL1, FTH1) or mitochondrial extracts (ALAS2) prepared from mouse liver. Protein levels were normalized to beta tubulin or NDUFB8, respectively. Protein data from one representative time series are shown (*N* = 2–3).

More than one-third (682/1874) of genes that cycled at the footprint level did not have a rhythmic mRNA (Fig. 4A); globally, these transcripts showed decreased TEs (location shift of −0.105 corresponds to a 7% TE reduction; *P* = 0.002) (Fig. 4H, light blue). However, closer inspection of the underlying RPF-seq and RNA-seq profiles indicated that in many cases, nonrhythmicity assignments at the mRNA level had resulted from noise or low amplitudes (close to the imposed 1.5-fold cut-off) in expression profiles that otherwise still appeared to be rhythmic (false-negatives caused by “cliff effects”) (Supplemental Fig. S6C). To refine the “mRNA flat–footprints rhythmic” assignments, we used the analytical framework *Babel* (Olshen et al. 2013) to first identify all transcripts that had significant TE differences over timepoints (and/or whose TEs significantly deviated from the global transcript population), and subsequently performed the rhythmicity analyses on these. This strategy resulted in a high-confidence set of 147 rhythmically translated, but otherwise nonoscillating, mRNAs (Fig. 5A; Supplemental Table S3). Their RPF-seq profiles showed a striking phase distribution with a dominant peak around the day-to-night transition (ZT10–ZT16) (Fig. 5B). Gene ontology (GO) analyses revealed enrichment for mRNAs encoding components of the protein biosynthesis machinery, including RPs, elongation factors, and poly(A) binding proteins, whose translation underwent a characteristic upsurge starting from ZT10 (Fig. 5C,D). Of note, increased polysome association at the beginning of the night has previously been described for this class of transcripts (Jouffe et al. 2013), which all contain 5′-TOP motifs that are regulated by TORC1 (Meyuhas and Kahan 2015). Of 79 RPs, 35 were contained in the high-confidence list, and—with the exception of eight proteins whose mRNAs were undetectable or translationally invariable—visual inspection confirmed that most other RPs shared a similar RPF-seq profile as well (Supplemental Fig. S7A; Supplemental Dataset 1). In summary, our data extend previous findings (Jouffe et al. 2013) and precisely quantify the coordination of protein biosynthesis within the translational apparatus in mouse liver. Of note, our study uncovers a peculiarity of RP gene expression, i.e., particularly high mRNA abundances paired with low TEs, which undergo coordinated upsurge/translational de-repression prior to the day-to-night transition (Supplemental Fig. S7B; Supplemental Movie M1).

Other GO terms and individual “mRNA flat–footprints rhythmic” transcripts caught our attention as well. The electron transport chain components that the GO analysis identified (Fig. 5C) corresponded throughout to mitochondrially encoded transcripts characterized by a translational spike at ZT12 (Supplemental Fig. S8A; Supplemental Dataset 1). As Western blot analysis did not reveal any oscillations at the protein level (Supplemental Fig. S8B), the significance of these translational rhythms remains to be uncovered. We next verified for other examples of rhythmic translation whether protein abundances oscillated. This was indeed the case for geranylgeranyl diphosphate (GGPP) synthase 1 (*Ggps1*), encoding a key branchpoint enzyme in the mevalonate pathway (Supplemental Fig. S8C,D). GGPP is important for the C20-prenylation of proteins and for the regulation of the nuclear receptor NR1H3/LXR alpha (Forman et al. 1997). Moreover, two transcription factors, Deformed epidermal autoregulatory factor 1 (*Deaf1*) and Max interactor 1 (*Mxi1*), showed robust greater than twofold rhythms in translation (Fig. 5E; Supplemental Fig. S8E). For DEAF1, steady-state protein levels oscillated as well (Fig. 5E), raising the interesting possibility that translational rhythmicity is propagated to transcriptional target genes and thus contributes to shaping the rhythmic transcriptome. In accordance, previously reported DEAF1 targets (Yip et al. 2009) were significantly enriched for genes that are rhythmically transcribed (enrichment 1.6-fold; *P* = 0.009) (Fig. 5F; identified by Du et al. 2014). Notably, the majority of DEAF1 target gene pre-mRNAs peaked between ZT6 and ZT12, coinciding with maximal DEAF1 protein abundance (Fig. 5E,F).

Finally, our data revealed that the well-known case of translational control through iron-responsive elements (IREs) undergoes high-amplitude oscillations. IREs are stem–loops found in mRNAs involved in iron, oxygen, and energy metabolism (see Anderson et al. 2012 and references therein). Depending on the cytosolic iron concentration and other cues (see Discussion), IREs located in 5′ or 3′ UTRs regulate mRNA translation and degradation, respectively. Ferritin heavy and light chain 1 (*Fth1, Ftl1*), involved in iron storage, as well as aminolevulinic acid synthase 2 (*Alas2*), a rate-limiting enzyme of heme synthesis, showed high-amplitude oscillations in translation that led to protein rhythmicity (Fig. 5G). All three transcripts contain IREs in their 5′ UTRs, whereas transferrin receptor (*Tfrc*) is a case where a 3′ UTR–borne IRE controls mRNA turnover (Anderson et al. 2012). *Tfrc* mRNA (and footprint) profiles were rhythmic in our data set (Supplemental Dataset 1); moreover, previous RNA-seq data quantifying pre-mRNA and mRNA levels around the clock (Du et al. 2014) revealed that the abundance of *Tfrc* mRNA, but not of its pre-mRNA (and hence, its transcription), oscillated (Supplemental Fig. S8F). These analyses suggest that regulation by IREs is overall under time of day–dependent control. Of note, besides the temporal regulation of IRE-containing transcripts, we observed high-amplitude oscillations in gene expression (RNA-seq and RPF-seq) throughout key steps in iron metabolism (Supplemental Fig. S8G). These findings indicated widespread rhythmic regulation of iron homeostasis that has been largely overlooked so far.

### Core clock transcripts show a broad range of TEs and abundant uORF usage

Although core clock transcript TEs were temporally invariable (see above) (Fig 3B; Supplemental Fig. S5A), further analyses of the data revealed features of regulation that were potentially of functional importance. First, we noticed that the intrinsic TEs of clock mRNAs spanned a greater than sixfold range from the poorest (*Clock, Per3*) to the best (*Nr1d1*) translators (Supplemental Fig. S5A,B, upper panel). TEs and mRNA abundances together define the amounts of protein that are produced, and RPF-seq RPKMs are hence a direct readout of relative protein biosynthesis levels. We therefore used our footprint counts to precisely quantify the stoichiometry at which the clock proteins are produced. Importantly, these estimates explained known features of the clock better than did RNA expression data alone. Notably, *Clock* is in excess of its heterodimerization partner *Arntl* at the transcript level (integrated over the day, about 1.6-fold more *Clock* than *Arntl* mRNA), but due to TE differences, about 1.5-fold more ARNTL than CLOCK protein is produced (Supplemental Fig. S5B; for similar results obtained when peak levels rather than daily amounts were considered, see Supplemental Fig. S5C), which is consistent with the conjecture that ARNTL is in excess over CLOCK (Huang et al. 2012). For the main positive and negative limb components, our data indicated daily biosynthesis at a ratio of CLOCK(1.0):ARNTL(1.5):PER1(0.6):PER2(1.1):CRY1(2.3):CRY2(2.3), i.e., overall similar levels of produced proteins (Supplemental Fig. S5B). Finally, it is established that in the interconnecting limb NR1D1/REV-ERB alpha represents the dominant *Rev-erb* paralog in liver (Preitner et al. 2002; Bugge et al. 2012). While *Nr1d2/Rev-erb beta* is nevertheless significantly more abundant at the mRNA level, the amount of biosynthesized NR1D1 protein exceeds the NR1D2 paralog by greater than twofold due to greater than fivefold differences in TEs (Supplemental Fig. S5B, lower panel). In summary, these analyses suggest that TE is an important factor in establishing the appropriate clock protein output. Conceivably, it may represent an additional layer at which the core clock can undergo regulation.

**Figure 6.**
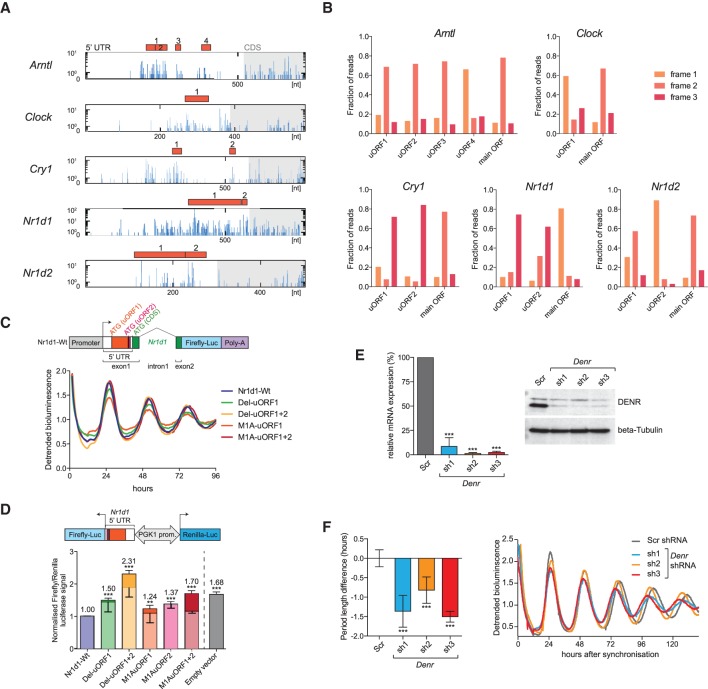
uORF translation is prevalent in core clock transcripts and impacts clock functions. (*A*) The 5′ UTRs of the depicted clock transcripts all contained at least one translated AUG-initiated uORF. Distribution of raw read counts of RPF reads (blue) along the 5′ UTR (white region in box) and the first 200 nt of the CDS (gray shaded region) is shown for the timepoint with maximal CDS translation. Red filled boxes indicate AUG-initiated uORFs within the 5′ UTR. Predicted uORFs for each gene are serially numbered. (*B*) Frame preference of uORF-mapping footprints. The fractions of footprints aligning to the three reading frames are shown for the uORFs shown in *A* and for the main ORF (CDS). Frame definition is relative to the annotated 5′ end of the transcript; please note that frame definition is different from that in Figure 1F. Most uORFs are thus covered by footprints that have a similar degree of frame preference as the main ORF-mapping footprints, indicating that uORF-mapping reads likely originate from processive translation. (*C*, *top*) Schematic showing the wild-type *Nr1d1*-firefly luciferase (FL) reporter gene consisting of a genomic *Nr1d1* fragment in which FL (blue) is expressed in fusion with the first 15 amino acids of NR1D1 (green). In exon 1, the location of uORF1 and -2 (red) and their predicted start codons within the 5′ UTR is shown. (*Bottom*) Real-time bioluminescence recordings of luciferase rhythms in NIH3T3 cells lentivirally transduced with the *Nr1d1*-FL reporter (Wt) and various mutants in which either uORFs are deleted (Del mutants) or uORF initiation codons are mutated to an alanine codon (M1A mutants). Cells were synchronized with dexamethasone. Raw bioluminescence was detrended using a 24-h moving average, and one representative replicate of a total *N* = 3–9 is shown. (*D*, *top*) Schematic representation of the dual luciferase reporter construct to measure how the *Nr1d1* 5′ UTR (Wt/mutants) influences the expression of the FL CDS. From the same bidirectional promoter, *Renilla* luciferase (RL) is expressed for internal control. (*Bottom*) Results of dual luciferase assay where FL signals were internally normalized to RL. Empty vector (gray) only contained the vector-encoded 5′ UTR. Experiments were performed in NIH3T3 cells (*N* = 2–4 independent experiments of triplicates). Lighter shading of the bars indicates the proportion of the increase that can be attributed to increased FL mRNA abundance in the mutants (measured by qRT-PCR), leaving the remainder of the increase (darker shading) attributable to translation. Note that whenever the translated uORF1 and -2 of the *Nr1d1* 5′ UTR are deleted (Del mutants) or just the initiation codons are mutated to alanine codons (M1A mutants), the inhibitory activity of the *Nr1d1* 5′ UTR is relieved. uORF1 and uORF2 appear to have an additive inhibitory effect on main ORF translation (cf. M1A uORF1 and -2 single mutants with the double mutant). For a schematic of the mutants, see also Supplemental Figure S9C. (*E*, *left*) Relative mRNA levels (RT-qPCR; normalized to expression of control gene *Nudt4*) of *Denr* in DBP-Luciferase reporter–expressing NIH3T3 cells transduced with scramble shRNA (Scr; serving as control) or three different shRNAs targeting *Denr* (*N* = 3). All shRNAs reduced *Denr* expression to <10%. (*Right*) Western blot analysis for DENR indicated efficient depletion at the protein level. Beta tubulin served as loading control. (*F*) shRNA-mediated *Denr* knockdown causes a short period phenotype of free-running circadian rhythms in NIH3T3 cells*.* (*Left*) Summary of period change engendered by *Denr* shRNAs 1–3 relative to Scr (control) shRNA in cells expressing the *DBP-Luciferase* reporter (*N* = 3–8). (*Right*) Representative bioluminescence tracks of Scr (control) and *Denr* shRNA-transduced DBP-Luciferase cells. Depending on the shRNA, the period of free-running circadian oscillations was 0.8–1.5 h shorter. (*D*–*F*) Bar graphs, mean ± SD; (**) *P* < 0.01, (***) *P* < 0.001 (*t*-test). *P*-value in *D* refers to differences in FL/RL activities (darker shading).

The striking correlation of uORF usage with TEs (Fig. 2C) prompted us to explore whether translated uORFs were present in core clock transcripts. Intriguingly, *Arntl, Clock, Cry1, Nr1d1*, and *Nr1d2,* all showed considerable ribosome occupancy in their 5′ UTRs and contained one or more AUG-initiated uORFs (Fig. 6A) with footprint coverage that showed clear frame preference, indicative of their active translation (Fig. 6B). To investigate how uORFs regulated the rhythmic production of a clock protein, we chose *Nr1d1*, for which RPF-seq reads on uORFs 1 and 2 showed particularly high coverage (Fig. 6A), frame bias (Fig. 6B), and rhythmicity in sync with the main ORF (Supplemental Fig. S9A). Moreover, uORF1 was remarkably long (192 nt) and conserved in mammals, potentially coding for a 63-amino-acid polypeptide (Supplemental Fig. S9B). We constructed a lentiviral reporter gene from an *Nr1d1* genomic fragment that contained promoter sequences conferring rhythmic transcription (Stratmann et al. 2010), exon 1 (5′ UTR and codons 1–10), intron 1, and a modified exon 2, in which firefly luciferase (FL) was fused to NR1D1 after amino acid 15 (Fig. 6C). Moreover, we designed reporter variants in which the predicted uORF initiation codons were mutated or the uORFs were deleted altogether (Supplemental Fig. S9C). When expressed in a circadian model cell line, NIH3T3 fibroblasts, all constructs showed comparable high-amplitude bioluminescence rhythms in the detrended data (Fig. 6C), indicating that uORFs were dispensable for rhythmic protein expression per se. Importantly, the differences in absolute luciferase signals that we observed between reporters (Supplemental Fig. S9D,E) could have resulted from altered TEs or simply reflect unequal lentiviral titers, transduction efficiencies, cell numbers, or similar. We therefore measured the effect of the uORFs on main ORF translation in an independent assay in which the 5′ UTRs were cloned upstream of FL CDS in a lentiviral vector that also expressed an internal control gene, *Renilla* luciferase (RL), driven from the same bidirectional promoter (Fig. 6D, top; Du et al. 2014). These analyses revealed that uORF deletions, or the subtle initiation codon mutations, led to increased levels of FL reporter activity (Fig. 6D, bottom). Moreover, uORF1 and uORF2 had an additive effect, as judged by mutants in which uORF1 and uORF2 AUGs were mutated to alanine codons either singly (mutants M1A-uORF1 and M1A-uORF2) or in combination (M1A-uORF1+2). We next measured RNA expression levels of both luciferases, which allowed us to estimate the relative contributions that altered RNA stability (lighter shading in Fig. 6D, bottom) and translation regulation (darker shading in Fig. 6D, bottom) made to the observed increases in reporter protein output. These analyses suggested dual contribution by both mechanisms, in line with the initial observation (Fig. 2C) of decreased TE and mRNA abundance of uORF-containing transcripts (the latter possibly involving regulation through the NMD pathway). Extrapolated to the regulation of the endogenous *Nr1d1* transcript in vivo, the prediction from these results would be that uORF1+2 could regulate the magnitude of NR1D1 oscillations.

There was evidence for translated uORFs in several core clock transcripts (Fig. 6A,B), but the net regulatory effect of uORF translation on clock function would likely be difficult to predict from studying each case individually. In order to estimate the overall impact of uORF translation on the clock, we took advantage of the recent discovery that Density Regulated Protein (DENR) is implicated in ribosome recycling after translation termination and acts as a selective regulator of reinitiation at the main CDS after uORF usage (Skabkin et al. 2013; Schleich et al. 2014). In the absence of DENR, proteins whose expression is regulated by uORFs are thus produced at lower levels (Schleich et al. 2014). We down-regulated endogenous *Denr* in NIH3T3 cells carrying a circadian *Dbp-Luciferase* reporter gene (Stratmann et al. 2012) using three different shRNAs (Fig. 6E). *Denr*-deficient cells showed robust period shortening of free-running circadian oscillations by up to 1.5 h (Fig. 6F); moreover, a short period phenotype was also observed using a second reporter, *Arntl-Luciferase* (Supplemental Fig. S9F). We concluded that DENR has important functions in the regulation of the mammalian circadian clock.

## Discussion

How translation efficiency contributes to temporal gene expression is a largely unexplored facet of chronobiology. Translation is one step closer than the mRNA to the relevant output of most gene expression, the protein. Our ribosome profiling data should therefore be of wide interest to the research community and complement the many transcriptome data sets that are already available. By providing a resource consisting of transcriptome-wide RPF-seq/RNA-seq and TE plots (Supplemental Dataset 1), associated rhythmicity parameters (Supplemental Table S2), and high-confidence transcript lists from *Babel* analysis (Supplemental Table S3), we wish to facilitate the widespread use of our data. This resource provides a number of straightforward opportunities for exciting future endeavors, one example being the interesting cases of genes whose mRNAs, but not the footprint profiles, oscillate (Fig. 4A; Supplemental Fig. S6C), which we were unable to further investigate within the scope of this study.

Our study provides part of the explanation for the longstanding enigma that the mRNAs of many oscillating proteins show constant abundance over the day (Reddy et al. 2006). Two recent reports have estimated that 20% (Robles et al. 2014) to 50% (Mauvoisin et al. 2014) of protein rhythms are engendered by translation, protein degradation, or secretion. Our high-confidence set of approximately 150 nonoscillating mRNAs that undergo robust daily TE rhythms corresponds to ∼8% of all detected rhythmically biosynthesized proteins. Considering the conservative selection criteria that were applied, the true extent of translationally driven rhythmicity may even be higher.

How does the translatome data correlate with the rhythmic proteome? The answer to this question is less straightforward than expected. A first complication lies in the poor overlap of the proteomics studies; although both report on almost 200 rhythmic proteins (Mauvoisin et al. 2014; Robles et al. 2014), <20% are shared between the studies, and the particularly interesting “mRNA flat–protein rhythmic” class has only three proteins in common (Supplemental Fig. S10A,B). As the overlap in the total detected proteome (about 3000–5000 polypeptides in both studies) is >50%, the differences seen in the rhythmic sets are not just a matter of proteome coverage. Differences in mass-spectrometric and, very likely, rhythmicity detection methodology may have caused the discrepancies; sophisticated meta-analyses on all available raw data using comparable algorithms and statistical parameters would thus be of great value. Another concern when comparing RPF-seq and proteome data sets is that MS data are inevitably biased for abundant (i.e., highly expressed and/or stable) polypeptides, whereas rhythmic TEs predominantly affected mRNAs whose abundance was below average (Fig. 4H). Many translationally regulated transcripts are hence not covered by the proteome data. It is reassuring that, despite such limitations, several translationally rhythmic transcripts are part of the rhythmic proteome (e.g., FTH1, EEF1A1, and EEF2 in study by Robles et al. 2014).

Transcripts encoding components of the protein biosynthesis machinery stand out among the rhythmically translated mRNAs. Their preferential association with polysomes at the light-to-dark transition has been reported before; it likely gates the energy-consuming ribosome biogenesis to the appropriate time when nutrients are plentiful, and involves regulatory cues from both feeding (via TORC1-regulated 5′-TOP motifs) and from the clock (Jouffe et al. 2013). As we observed increased TEs on these mRNAs already at ZT10 (Fig. 5D), i.e., ∼2 h before the main surge in food intake in ad libitum fed animals (Adamovich et al. 2014), we consider it likely that the mechanism entails more than a simple, immediate reaction to nutrients. Moreover, the relatively variable up-regulation seen across biological replicates (Fig. 5D, RPF-seq error bars) is remarkable for genetically identical animals and could point to a behavioral component contributing to the regulatory mechanism. Interestingly, and reminiscent of the timing in the liver, increased ribosome association of mRNAs in *Drosophila* occurs at phases of relative behavioral quiescence, just prior to locomotor activity bouts (Huang et al. 2013). It remains to be explored whether this similarity is indicative of mechanistic parallels. Another exciting open question concerns the possibility that the rhythmic biosynthesis of components of the translational apparatus contributes to daily changes in overall translation rate that have been reported and that may involve mTOR signaling and a noncanonical cytoplasmic role for ARNTL (Lipton et al. 2015).

Among the other cases of TE rhythmicity, only a few were directly suggestive of an underlying mechanism, as was the case for mRNAs encoding iron metabolic proteins that all contain IREs. IREs are bound by iron regulatory proteins (IRPs) 1 and 2 (encoded by the genes *Aco1* and *Ireb2*, respectively), which sense intracellular iron levels by distinct mechanisms and respond to other metabolic signals as well (for review, see Anderson et al. 2012). IRP1 assembles a 4Fe-4S cluster in response to increased iron availability, which precludes IRE binding and permits translation. Other signals, such as NO, H_2_O_2_, and O_2_, also influence the Fe-S cluster and IRP1 activity. IRP2 is regulated by protein degradation via FBXL5, an E3 ubiquitin ligase stabilized by iron and oxygen. We did not observe high-amplitude rhythms in the expression of IRPs or their known regulators (Supplemental Fig. S11; please note low amplitude rhythms for *Ireb2*), and it is conceivable that rhythmicity occurs at the level of available, bioactive iron in the hepatocyte (day/night changes in hepatic total iron have been reported) (Unger et al. 2009), of O_2_ pressure/consumption (Peek et al. 2013), or of reactive oxygen species (Khapre et al. 2011). Together with the oscillations in mRNA abundance seen for multiple other iron metabolic genes, the rhythmic regulation of IRE-containing transcripts uncovers a previously unappreciated extent of temporal control in this physiologically important pathway.

It is noteworthy that clock genes showed constant TEs, indicating exclusion from time of day–dependent translational control. The considerable delays between mRNA and protein accumulation that have been reported for several core clock components (e.g., Lee et al. 2001) must therefore have other, post-translational origins. Nevertheless, our study has unveiled important insights into how translation contributes to core clock regulation. First, the CDS-mapping RPF-seq reads allow estimating relative biosynthesis rates of core clock proteins, which will likely add to a better quantitative understanding of the clock mechanism. Moreover, the footprint profiles from several clock mRNAs showed hallmarks of regulation that, however, may be operative not in a temporal fashion but under other (e.g., environmental, metabolic, cell-type–specific) conditions yet to be defined. In this context, the high number of translated uORFs within the core clock transcripts is particularly striking. uORF translation is generally viewed as inhibitory for protein production from the main ORF (Wethmar et al. 2014) and represents an attractive mechanism for how clock protein levels (and consequently clock parameters) could be adjusted post-transcriptionally. It is tempting to speculate that one or several of the identified core clock uORFs are implicated in the short period phenotype observed in *Denr*-depleted cells. Of note, there is growing evidence for cell-type–specific uORF usage (e.g., Ingolia et al. 2011), and it is also largely unexplained how certain core clock parameters can be strikingly tissue specific (e.g., >2 h longer free-running period in kidney vs. lung) (Yoo et al. 2004). It is conceivable that cell-type–specific differences in clock protein concentration and/or stoichiometry are involved (Lee et al. 2011) and that tissue-specific uORF usage and translation rates contribute. Altogether, our results suggest that the circadian system represents a particularly suitable paradigm for future studies of uORF biology.

## Methods

### Animals

For time series experiments, 12-wk-old male mice (C57BL/6J; Janvier Labs) were entrained for 2 wk to LD 12:12 with free access to food and water and were euthanized at indicated *Zeitgeber* times (ZT0 corresponding to “lights on”) by decapitation after anesthesia (isoflurane). Livers were removed and processed either directly or flash-frozen in liquid N_2_. All experimental procedures were approved by the Veterinary Office of the Canton Vaud (authorization VD2376).

### Ribosome profiling and RNA-seq

RPF-seq and RNA-seq libraries were generated using Ribo-Zero and ARTseq ribosome profiling kits (Epicentre) and sequenced on an Illumina HiSeq 2500. Detailed protocols, including for lysate preparation, are described in the Supplemental Material.

### Bioinformatic analysis of ribosome profiling and RNA-seq

Adapter-trimmed, size-filtered sequencing reads (lengths 26–35 nt and 21–60 nt for RPF-seq and RNA-seq, respectively) were mapped sequentially to mouse rRNA, human rRNA, mt-tRNA, mouse tRNA, mouse cDNA (Ensembl release 75), and, finally, mouse genomic sequences (GRCm38.p2). cDNA-mapping reads were counted toward 5′ UTR, CDS, and 3′ UTR per gene basis. CDS counts were normalized by the upper quantile method and transformed into modified RPKM values. TEs were calculated as the ratio of RPF-RPKM to mRNA-RPKM. For detailed information on bioinformatic analysis, see the Supplemental Material.

### Protein analyses

Total, nuclear, and mitochondrial protein extracts were prepared from two to three individual mice per timepoint and analyzed by SDS-PAGE and Western blotting according to standard protocols. Figures show one representative time series. Detailed experimental protocols and antibodies are described in the Supplemental Material.

### Cloning

The generation of lentiviral luciferase reporter plasmids containing wild-type/mutant fragments of the *Nr1d1* genomic region is described in the Supplemental Material. For the generation of lentiviral shRNA expression vectors targeting *Denr*, sequences from the TRC shRNA Library at the Broad Institute were cloned into pLKO.1puro backbone vector (Addgene no. 10878) (Moffat et al. 2006); sequences/clones are listed in Supplemental Material. Scramble shRNA (Addgene no. 1864) served as control.

### Cell culture

Cell culture, lentiviral production/transduction, the recording of circadian bioluminescence rhythms, and the dual luciferase assays followed standard methods. Detailed experimental protocols and additional references can be found in the Supplemental Material.

### RT-qPCR

RNA was extracted with TriFast peqGOLD (PEQLAB), reverse-transcribed with SuperScript II (Invitrogen), and amplified with SYBR green rox master mix (Roche) and gene-specific primers (Supplemental Material) on a Stratagene Mx3000P apparatus (Agilent). Relative expression levels were determined using the ΔΔCt method.

### GO analysis

GO analysis was carried out on the footprints rhythmic set after Babel analysis (Olshen et al. 2013) using the DAVID bioinformatics resource (Huang et al. 2009).

## Data access

The sequencing data from this study have been submitted to the NCBI Gene Expression Omnibus (GEO; http://www.ncbi.nlm.nih.gov/geo/) under accession number GSE67305.

## References

[JANICHGR195404C1] Adamovich Y, Rousso-Noori L, Zwighaft Z, Neufeld-Cohen A, Golik M, Kraut-Cohen J, Wang M, Han X, Asher G. 2014 Circadian clocks and feeding time regulate the oscillations and levels of hepatic triglycerides. Cell Metab 19: 319–330. 24506873 10.1016/j.cmet.2013.12.016 PMC4261230

[JANICHGR195404C2] Anderson CP, Shen M, Eisenstein RS, Leibold EA. 2012 Mammalian iron metabolism and its control by iron regulatory proteins. Biochim Biophys Acta 1823: 1468–1483. 22610083 10.1016/j.bbamcr.2012.05.010 PMC3675657

[JANICHGR195404C3] Arava Y, Wang Y, Storey JD, Liu CL, Brown PO, Herschlag D. 2003 Genome-wide analysis of mRNA translation profiles in *Saccharomyces cerevisiae*. Proc Natl Acad Sci 100: 3889–3894. 12660367 10.1073/pnas.0635171100 PMC153018

[JANICHGR195404C4] Bugge A, Feng D, Everett LJ, Briggs ER, Mullican SE, Wang F, Jager J, Lazar MA. 2012 Rev-erbα and Rev-erbβ coordinately protect the circadian clock and normal metabolic function. Genes Dev 26: 657–667. 22474260 10.1101/gad.186858.112 PMC3323877

[JANICHGR195404C5] Churbanov A, Rogozin IB, Babenko VN, Ali H, Koonin EV. 2005 Evolutionary conservation suggests a regulatory function of AUG triplets in 5′-UTRs of eukaryotic genes. Nucleic Acids Res 33: 5512–5520. 16186132 10.1093/nar/gki847 PMC1236974

[JANICHGR195404C6] Dana A, Tuller T. 2012 Determinants of translation elongation speed and ribosomal profiling biases in mouse embryonic stem cells. PLoS Comput Biol 8: e1002755. 23133360 10.1371/journal.pcbi.1002755 PMC3486846

[JANICHGR195404C7] Du NH, Arpat AB, De Matos M, Gatfield D. 2014 MicroRNAs shape circadian hepatic gene expression on a transcriptome-wide scale. eLife 3: e02510. 24867642 10.7554/eLife.02510 PMC4032493

[JANICHGR195404C8] Forman BM, Ruan B, Chen J, Schroepfer GJ Jr, Evans RM. 1997 The orphan nuclear receptor LXRα is positively and negatively regulated by distinct products of mevalonate metabolism. Proc Natl Acad Sci 94: 10588–10593. 9380679 10.1073/pnas.94.20.10588 PMC23411

[JANICHGR195404C9] Huang DW, Sherman BT, Lempicki RA. 2009 Systematic and integrative analysis of large gene lists using DAVID bioinformatics resources. Nat Protoc 4: 44–57. 19131956 10.1038/nprot.2008.211

[JANICHGR195404C10] Huang N, Chelliah Y, Shan Y, Taylor CA, Yoo SH, Partch C, Green CB, Zhang H, Takahashi JS. 2012 Crystal structure of the heterodimeric CLOCK:BMAL1 transcriptional activator complex. Science 337: 189–194. 22653727 10.1126/science.1222804 PMC3694778

[JANICHGR195404C11] Huang Y, Ainsley JA, Reijmers LG, Jackson FR. 2013 Translational profiling of clock cells reveals circadianly synchronized protein synthesis. PLoS Biol 11: e1001703. 24348200 10.1371/journal.pbio.1001703 PMC3864454

[JANICHGR195404C12] Ingolia NT. 2014 Ribosome profiling: new views of translation, from single codons to genome scale. Nat Rev Genet 15: 205–213. 24468696 10.1038/nrg3645

[JANICHGR195404C13] Ingolia NT, Ghaemmaghami S, Newman JR, Weissman JS. 2009 Genome-wide analysis in vivo of translation with nucleotide resolution using ribosome profiling. Science 324: 218–223. 19213877 10.1126/science.1168978 PMC2746483

[JANICHGR195404C14] Ingolia NT, Lareau LF, Weissman JS. 2011 Ribosome profiling of mouse embryonic stem cells reveals the complexity and dynamics of mammalian proteomes. Cell 147: 789–802. 22056041 10.1016/j.cell.2011.10.002 PMC3225288

[JANICHGR195404C15] Jouffe C, Cretenet G, Symul L, Martin E, Atger F, Naef F, Gachon F. 2013 The circadian clock coordinates ribosome biogenesis. PLoS Biol 11: e1001455. 23300384 10.1371/journal.pbio.1001455 PMC3536797

[JANICHGR195404C16] Khapre RV, Kondratova AA, Susova O, Kondratov RV. 2011 Circadian clock protein BMAL1 regulates cellular senescence in vivo. Cell Cycle 10: 4162–4169. 22101268 10.4161/cc.10.23.18381 PMC3272294

[JANICHGR195404C17] Kojima S, Sher-Chen EL, Green CB. 2012 Circadian control of mRNA polyadenylation dynamics regulates rhythmic protein expression. Genes Dev 26: 2724–2736. 23249735 10.1101/gad.208306.112 PMC3533077

[JANICHGR195404C18] Le Martelot G, Canella D, Symul L, Migliavacca E, Gilardi F, Liechti R, Martin O, Harshman K, Delorenzi M, Desvergne B, 2012 Genome-wide RNA polymerase II profiles and RNA accumulation reveal kinetics of transcription and associated epigenetic changes during diurnal cycles. PLoS Biol 10: e1001442. 23209382 10.1371/journal.pbio.1001442 PMC3507959

[JANICHGR195404C19] Lee C, Etchegaray JP, Cagampang FR, Loudon AS, Reppert SM. 2001 Posttranslational mechanisms regulate the mammalian circadian clock. Cell 107: 855–867. 11779462 10.1016/s0092-8674(01)00610-9

[JANICHGR195404C20] Lee Y, Chen R, Lee HM, Lee C. 2011 Stoichiometric relationship among clock proteins determines robustness of circadian rhythms. J Biol Chem 286: 7033–7042. 21199878 10.1074/jbc.M110.207217 PMC3044960

[JANICHGR195404C21] Lipton JO, Yuan ED, Boyle LM, Ebrahimi-Fakhari D, Kwiatkowski E, Nathan A, Guttler T, Davis F, Asara JM, Sahin M. 2015 The circadian protein BMAL1 regulates translation in response to S6K1-mediated phosphorylation. Cell 161: 1138–1151. 25981667 10.1016/j.cell.2015.04.002 PMC4447213

[JANICHGR195404C22] Mauvoisin D, Wang J, Jouffe C, Martin E, Atger F, Waridel P, Quadroni M, Gachon F, Naef F. 2014 Circadian clock-dependent and -independent rhythmic proteomes implement distinct diurnal functions in mouse liver. Proc Natl Acad Sci 111: 167–172. 24344304 10.1073/pnas.1314066111 PMC3890886

[JANICHGR195404C23] Mendell JT, Sharifi NA, Meyers JL, Martinez-Murillo F, Dietz HC. 2004 Nonsense surveillance regulates expression of diverse classes of mammalian transcripts and mutes genomic noise. Nat Genet 36: 1073–1078. 15448691 10.1038/ng1429

[JANICHGR195404C24] Meyuhas O, Kahan T. 2015 The race to decipher the top secrets of TOP mRNAs. Biochim Biophys Acta 1849: 801–811. 25234618 10.1016/j.bbagrm.2014.08.015

[JANICHGR195404C25] Moffat J, Grueneberg DA, Yang X, Kim SY, Kloepfer AM, Hinkle G, Piqani B, Eisenhaure TM, Luo B, Grenier JK, 2006 A lentiviral RNAi library for human and mouse genes applied to an arrayed viral high-content screen. Cell 124: 1283–1298. 16564017 10.1016/j.cell.2006.01.040

[JANICHGR195404C26] Mohawk JA, Green CB, Takahashi JS. 2012 Central and peripheral circadian clocks in mammals. Annu Rev Neurosci 35: 445–462. 22483041 10.1146/annurev-neuro-060909-153128 PMC3710582

[JANICHGR195404C27] Olshen AB, Hsieh AC, Stumpf CR, Olshen RA, Ruggero D, Taylor BS. 2013 Assessing gene-level translational control from ribosome profiling. Bioinformatics 29: 2995–3002. 24048356 10.1093/bioinformatics/btt533 PMC3834798

[JANICHGR195404C28] Peek CB, Affinati AH, Ramsey KM, Kuo HY, Yu W, Sena LA, Ilkayeva O, Marcheva B, Kobayashi Y, Omura C, 2013 Circadian clock NAD^+^ cycle drives mitochondrial oxidative metabolism in mice. Science 342: 1243417. 24051248 10.1126/science.1243417 PMC3963134

[JANICHGR195404C29] Preitner N, Damiola F, Lopez-Molina L, Zakany J, Duboule D, Albrecht U, Schibler U. 2002 The orphan nuclear receptor REV-ERBα controls circadian transcription within the positive limb of the mammalian circadian oscillator. Cell 110: 251–260. 12150932 10.1016/s0092-8674(02)00825-5

[JANICHGR195404C30] Reddy AB, Karp NA, Maywood ES, Sage EA, Deery M, O'Neill JS, Wong GK, Chesham J, Odell M, Lilley KS, 2006 Circadian orchestration of the hepatic proteome. Curr Biol 16: 1107–1115. 16753565 10.1016/j.cub.2006.04.026

[JANICHGR195404C31] Robles MS, Cox J, Mann M. 2014 In-vivo quantitative proteomics reveals a key contribution of post-transcriptional mechanisms to the circadian regulation of liver metabolism. PLoS Genet 10: e1004047. 24391516 10.1371/journal.pgen.1004047 PMC3879213

[JANICHGR195404C32] Schleich S, Strassburger K, Janiesch PC, Koledachkina T, Miller KK, Haneke K, Cheng YS, Kuchler K, Stoecklin G, Duncan KE, 2014 DENR–MCT-1 promotes translation re-initiation downstream of uORFs to control tissue growth. Nature 512: 208–212. 25043021 10.1038/nature13401 PMC4134322

[JANICHGR195404C33] Skabkin MA, Skabkina OV, Hellen CU, Pestova TV. 2013 Reinitiation and other unconventional posttermination events during eukaryotic translation. Mol Cell 51: 249–264. 23810859 10.1016/j.molcel.2013.05.026 PMC4038429

[JANICHGR195404C34] Stratmann M, Stadler F, Tamanini F, van der Horst GT, Ripperger JA. 2010 Flexible phase adjustment of circadian *albumin D site-binding protein* (*Dbp*) gene expression by CRYPTOCHROME1. Genes Dev 24: 1317–1328. 20551177 10.1101/gad.578810 PMC2885666

[JANICHGR195404C35] Stratmann M, Suter DM, Molina N, Naef F, Schibler U. 2012 Circadian *Dbp* transcription relies on highly dynamic BMAL1-CLOCK interaction with E boxes and requires the proteasome. Mol Cell 48: 277–287. 22981862 10.1016/j.molcel.2012.08.012

[JANICHGR195404C36] Unger EL, Earley CJ, Beard JL. 2009 Diurnal cycle influences peripheral and brain iron levels in mice. J Appl Physiol 106: 187–193. 18988764 10.1152/japplphysiol.91076.2008 PMC2636939

[JANICHGR195404C37] Vogel C, Marcotte EM. 2012 Insights into the regulation of protein abundance from proteomic and transcriptomic analyses. Nat Rev Genet 13: 227–232. 22411467 10.1038/nrg3185 PMC3654667

[JANICHGR195404C38] Vollmers C, Gill S, DiTacchio L, Pulivarthy SR, Le HD, Panda S. 2009 Time of feeding and the intrinsic circadian clock drive rhythms in hepatic gene expression. Proc Natl Acad Sci 106: 21453–21458. 19940241 10.1073/pnas.0909591106 PMC2795502

[JANICHGR195404C39] Wethmar K, Barbosa-Silva A, Andrade-Navarro MA, Leutz A. 2014 uORFdb: a comprehensive literature database on eukaryotic uORF biology. Nucleic Acids Res 42 (Database issue): D60–D67. 24163100 10.1093/nar/gkt952 PMC3964959

[JANICHGR195404C40] Yip L, Su L, Sheng D, Chang P, Atkinson M, Czesak M, Albert PR, Collier AR, Turley SJ, Fathman CG, 2009 Deaf1 isoforms control the expression of genes encoding peripheral tissue antigens in the pancreatic lymph nodes during type 1 diabetes. Nat Immunol 10: 1026–1033. 19668219 10.1038/ni.1773 PMC2752139

[JANICHGR195404C41] Yoo SH, Yamazaki S, Lowrey PL, Shimomura K, Ko CH, Buhr ED, Siepka SM, Hong HK, Oh WJ, Yoo OJ, 2004 PERIOD2::LUCIFERASE real-time reporting of circadian dynamics reveals persistent circadian oscillations in mouse peripheral tissues. Proc Natl Acad Sci 101: 5339–5346. 14963227 10.1073/pnas.0308709101 PMC397382

[JANICHGR195404C42] Zhang R, Lahens NF, Ballance HI, Hughes ME, Hogenesch JB. 2014 A circadian gene expression atlas in mammals: implications for biology and medicine. Proc Natl Acad Sci 111: 16219–16224. 25349387 10.1073/pnas.1408886111 PMC4234565

